# Evaluation of the Urine POC-CCA Test Accuracy in the Detection of *Schistosoma mansoni* Infection: A Systematic Review and Meta-Analysis

**DOI:** 10.1155/2024/5531687

**Published:** 2024-07-15

**Authors:** Getaneh Alemu, Endalkachew Nibret

**Affiliations:** ^1^ Department of Medical Laboratory Science Bahir Dar University, Bahir Dar, Ethiopia; ^2^ Biology Department Science College Bahir Dar University, Bahir Dar, Ethiopia; ^3^ Health Biotechnology Division Institute of Biotechnology (IoB) Bahir Dar University, Bahir Dar, Ethiopia

## Abstract

**Background:**

Schistosomiasis is a common public health problem throughout the world and *Schistosoma mansoni* is the most prevalent species in Africa. Most endemic countries use the Kato–Katz (KK) stool smear examination for diagnosis, mapping, and monitoring of intervention programs. However, its poor sensitivity calls for an urgency to evaluate and use more accurate diagnostic tools, of which detection of circulating cathodic antigen (CCA) in urine seems promising.

**Methods:**

Studies published until May 2022 were searched from PubMed, Google Scholar, and grey literature for systematic review and meta-analysis following the PRISMA guideline. Eligible studies were selected based on preset inclusion and exclusion criteria. Quality of included studies was assessed using the QUADAS-2 tool. Heterogeneity between studies was assessed using Cochrane *Q* test and *I*^2^ test statistics. Data were analyzed using Review Manager 5.4.1 and Meta-DiSc 1.4 software programs.

**Results:**

Thirty-seven studies published in 29 papers and enrolling 21159 study participants were included for analysis. Overall analysis of Point-of-Care Circulating Cathodic Antigen (POC-CCA) test against KK reference standard revealed a pooled sensitivity and specificity of 0.86 (95% CI: 0.85–0.87) and 0.66 (95% CI: 0.65–0.67), respectively. Subgroup analysis among 24 studies comparing single POC-CCA with test single KK revealed a high sensitivity (0.88) but low specificity (0.66). Based on findings of 24 studies, the area under the curve (AUC) for the systematic receiver operating characteristic (SROC) curve was 0.7805, indicating that the POC-CCA test effectively separates those with the disease from those who do not have it. Higher sensitivity estimates of 0.93 and 0.90 were reported when comparisons were made between test results of 2 urine and 1 stool samples, and 3 urine and 3 stool samples, respectively. Single POC-CCA test resulted in a pooled sensitivity estimate of 0.81 (95% CI: 0.78–0.84) as evaluated by the polymerase chain reaction (PCR) reference test.

**Conclusions:**

The POC-CCA test has higher sensitivity than KK and may serve as a routine diagnostic alternative for disease diagnosis, mapping, and monitoring of interventions. However, its accuracy should further be evaluated at different transmission settings and infection intensity.

## 1. Background

Schistosomiasis is a waterborne disease caused by blood-dwelling flukes of the genus *Schistosoma* [1]. *Schistosoma mansoni*, *S. japonicum*, and *S. haematobium* are the most common disease causing species [2]. The disease is endemic in 78 countries where 780 million people are at risk of infection. More than 250 million people are infected globally with more than 90% of the infections occurring in sub-Saharan Africa. In Africa, *S. mansoni* and *S. haematobium* are widespread causing intestinal and genitourinary schistosomiasis, respectively. Intestinal schistosomiasis poses most common public health problem throughout the continent. Schistosomiasis is a public health problem in Ethiopia where about 53.3 million people are at risk of infection and 4 million are already infected [3, 4]. Currently, two species are found in Ethiopia in the genus *Schistosoma*: *Schistosoma mansoni* is widespread throughout the country while *S. haematobium* has focal distribution in the low land borders of the country [5–7].

Intestinal schistosomiasis can be readily diagnosed using parasitological, immunological, and molecular techniques. The World Health Organization (WHO) recommends the Kato–Katz (KK) technique as the “gold standard” technique for screening of intestinal schistosomiasis due to its field applicability and possibility of quantitative reporting [8]. However, the KK thick smear is poorly sensitive especially in low transmission areas [9, 10]. More importantly, the ongoing mass drug administration (MDA) program is thought to decrease infection intensity and reduce worm fecundity that the egg output will be too low to be detected by the KK method. Daily variations in egg excretion and unisex infection also affect the test performance [11–13]. This calls for urgency in evaluation and use of new diagnostic methods which are more sensitive and affordable in resource-limited countries like Ethiopia. In recent years, antigen detection rapid diagnostic tests have sought great attention and both laboratory and field-based evaluations have been done at different geographical settings. Studies show that the urine Point-of-Care Circulating Cathodic Antigen (POC-CCA) test has superior performance as compared to KK [14, 15]. Other studies, on the contrary, reported that the CCA test has low sensitivity, especially in low transmission settings [16]. However, there is paucity of comprehensive data summarizing those findings. Therefore, considering that systematic reviews provide the best evidence for decision makers, we conducted a systematic review and meta-analysis on urine POC-CCA test accuracy in the diagnosis of infection by *S. mansoni*.

## 2. Methods

### 2.1. Study Settings

Studies conducted all over the world were included in the present review because we believe that geographical location has no direct effect in the performance of the POC-CCA test, rather level of transmission, test interpretation threshold, and number of samples examined mainly affect the accuracy.

### 2.2. Information Sources and Search of Literature

Potential articles were searched in PubMed, Google Scholar, and grey literature following the PRISMA guideline and checklist updated in 2020 [17]. Search in databases was done using the key terms: “Performance, accuracy, Circulating cathodic antigen, CCA, Kato Katz, Polymerase chain reaction (PCR), *Schistosoma mansoni*” combined with Boolean operators (AND, OR). A search in Google Scholar was made using the term “circulating cathodic antigen” in the title. The search and study selection was done from June 03 to August 16, 2022, by two reviewers independently.

### 2.3. Study Selection

**Figure 1:**
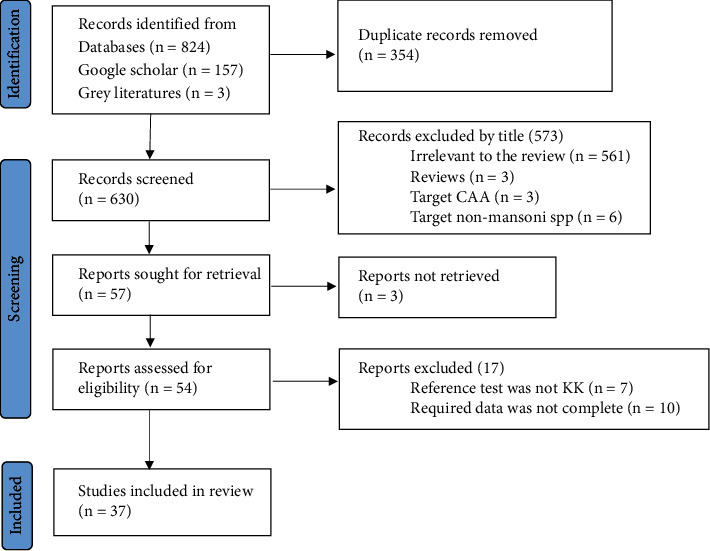
Flowchart showing selection process of eligible studies.

The study selection process is shown in Figure 1. Relevant studies were selected after sequential screening based on the title, abstract, and full text based on the following inclusion criteria: (1) institution or community-based studies published and available online until June 30, 2022; (2) studies targeting humans of any age group and published in English language; (3) studies of any design which compared urine POC-CCA as an index test with stool KK or with PCR reference tests; and (4) studies reporting the number of participants with true positive (TP), false positive (FP), false negative (FN), and true negative (TN) POC-CCA test results. Studies focusing on comparison of POC-CCA with other (than KK or PCR) diagnostic methods; studies presenting POC-CCA sensitivity and specificity without providing TP, FP, FN, and TN; studies targeting non-mansoni species; and reviews were excluded. Studies which did not explain the number of stool and urine samples examined for each participant were also excluded from the present review. Discrepancies in selection of studies between the two authors were resolved by discussion.

### 2.4. Data Extraction

Two authors independently extracted data using standard data collection form constructed in Excel sheet. Information was collected regarding the total sample size; age group of study participants; number of stool and urine samples collected and examined; separate number of participants with positive result by each test (POC-CCA, KK, and PCR); year of study; country of study; and number of TP, FP, FN, and TN test results by the index test (POC-CCA) as compared to the reference tests (KK or PCR).

### 2.5. Statistical Analysis

Pooled accuracy of urine POC-CCA test was analyzed against reference KK or PCR using Meta-DiSc 1.4 software. The number of participants with TP, FP, FN, and TN POC-CCA test results was used to calculate sensitivity and specificity of each study and an overall summary as well. Positive likelihood ratio (LR+), negative likelihood ratio (LR−), and diagnostic odds ratio (DOR) were also calculated. Subgroup analysis was done by number of stool and urine samples tested from each participant. In order to assess the ability of POC-CCA in discriminating participants with *S. mansoni* infection (TP rate) from noninfected (FP rate), summary receiver operating characteristic (SROC) curve was drawn and interpreted based on area under the curve (AUC) value as excellent (0.9-1.0), good (0.8-<0.9), fair (0.7-<0.8), poor (0.6-<0.7), and failed (0.5-<0.6) [18]. Heterogeneity between studies was checked with forest plot, Cochrane's *Q* test, and *I*^2^ test. Significant heterogeneity was declared at *I*^2^ > 50% and *Q*-test *p* value < 0.10. Methodological quality of the included studies was assessed by the Quality Assessment of Diagnostic Accuracy Studies 2 (QUADAS-2) tool using Review Manager 5.4.1 software.

## 3. Results

### 3.1. Characteristics of Included Studies

Thirty-seven studies published in 29 papers were included for analysis (Figure 1). A total of 21159 study participants were recruited in those studies. There was great variation among studies in terms of the age range of included studies with the majority (23 studies) enrolling school-aged children (SAC). Included studies were published between 2008 and 2021. Thirty studies were conducted in Africa [16, 19–39] while 6 were from Brazil [40–45]. One study was conducted in Switzerland, but the study participants were newly arriving Eritrean refuges [46]. A single stool and urine sample were collected and examined in 24 studies [16, 20, 21, 23, 24, 26, 28–30, 33–38, 42, 43, 45, 46]. Three publications [25, 26, 39] which separately analyzed POC-CCA accuracy with different number of urine samples were managed as 7 publications while one multicountry study [24] was treated as 5 publications (Table 1).

**Figure 2:**
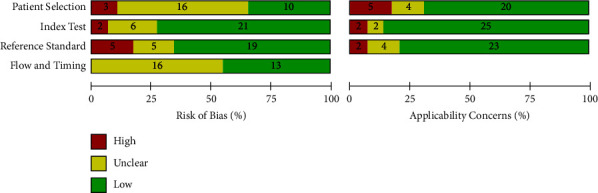
Risk of bias and applicability concerns graph.

Figure 2 shows the risk of bias and applicability concern results of the 29 included papers as analyzed by QUADAS-2 tool in Review Manager 5.4.1. The objective of doing this quality assessment is to assess the validity of estimates of POC-CCA test accuracy generated by this review and the applicability of included evidence to the review objective. Accordingly, except for the patient selection, nearly half or more of included studies got a score of low risk of bias. In majority of the studies, there was low applicability concern (Figure 2).

### 3.2. Accuracy of POC-CCA versus KK

**Figure 3:**
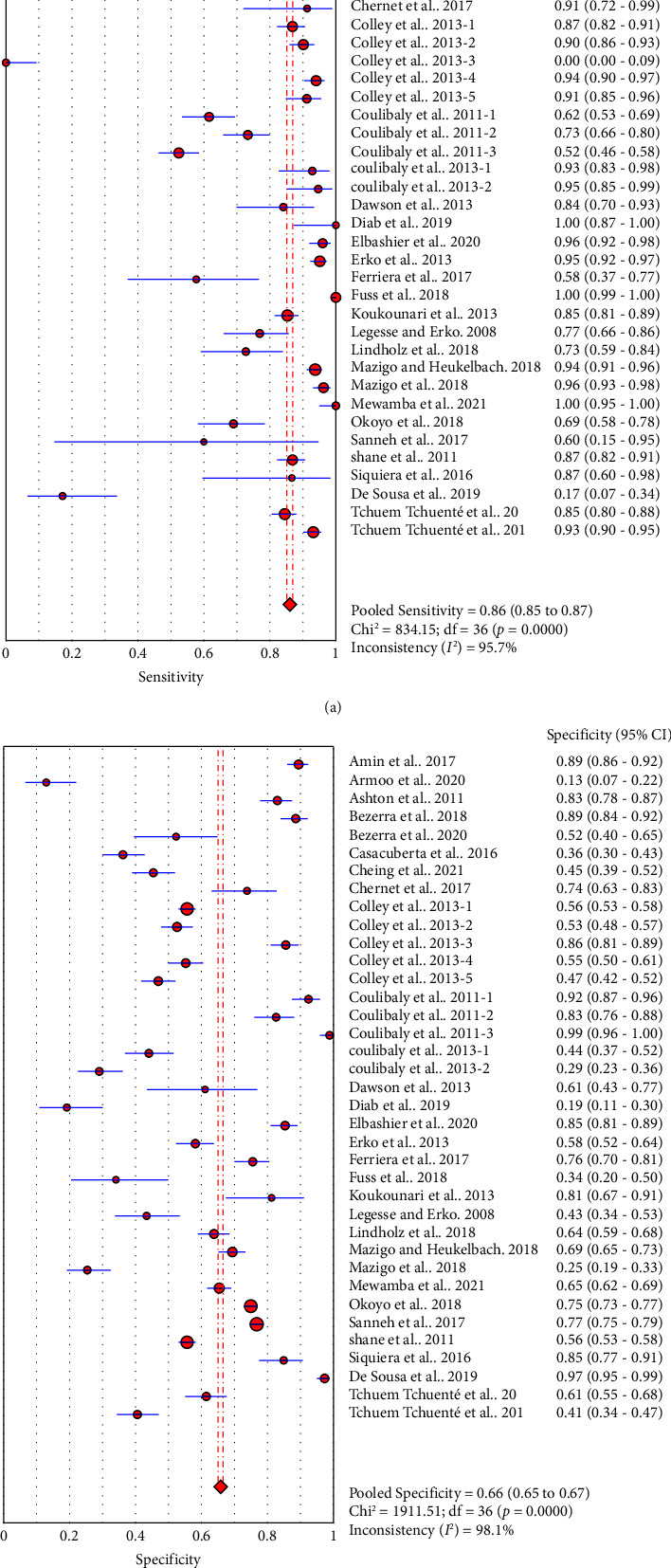
Sensitivity (a) and specificity (b) of POC-CCA compared to KK regardless of the number of samples examined (*n* = 37).

Thirty-seven studies compared accuracy of POC-CCA with KK. Overall analysis of POC-CCA against KK reference standard revealed a pooled sensitivity of 0.86 (95% CI, 0.85–0.87) while the specificity was 0.66 (95% CI, 0.65–0.67). The respective LR+ and LR− were 2.38 (95% CI 2.03–2.80) and 0.18 (95% CI: 0.10–0.33) while the DOR and AUC were 13.39 (95% CI: 9.27–19.34) and 0.82, respectively. The calculated AUC value implies a good performance of POC-CCA in discriminating *S. mansoni* infected people from noninfected ones. A random effect model was used in the analysis because included studies were heterogeneous (Figure 3).

### 3.3. Subgroup Analysis

#### 3.3.1. Single POC-CCA versus Single KK

**Figure 4:**
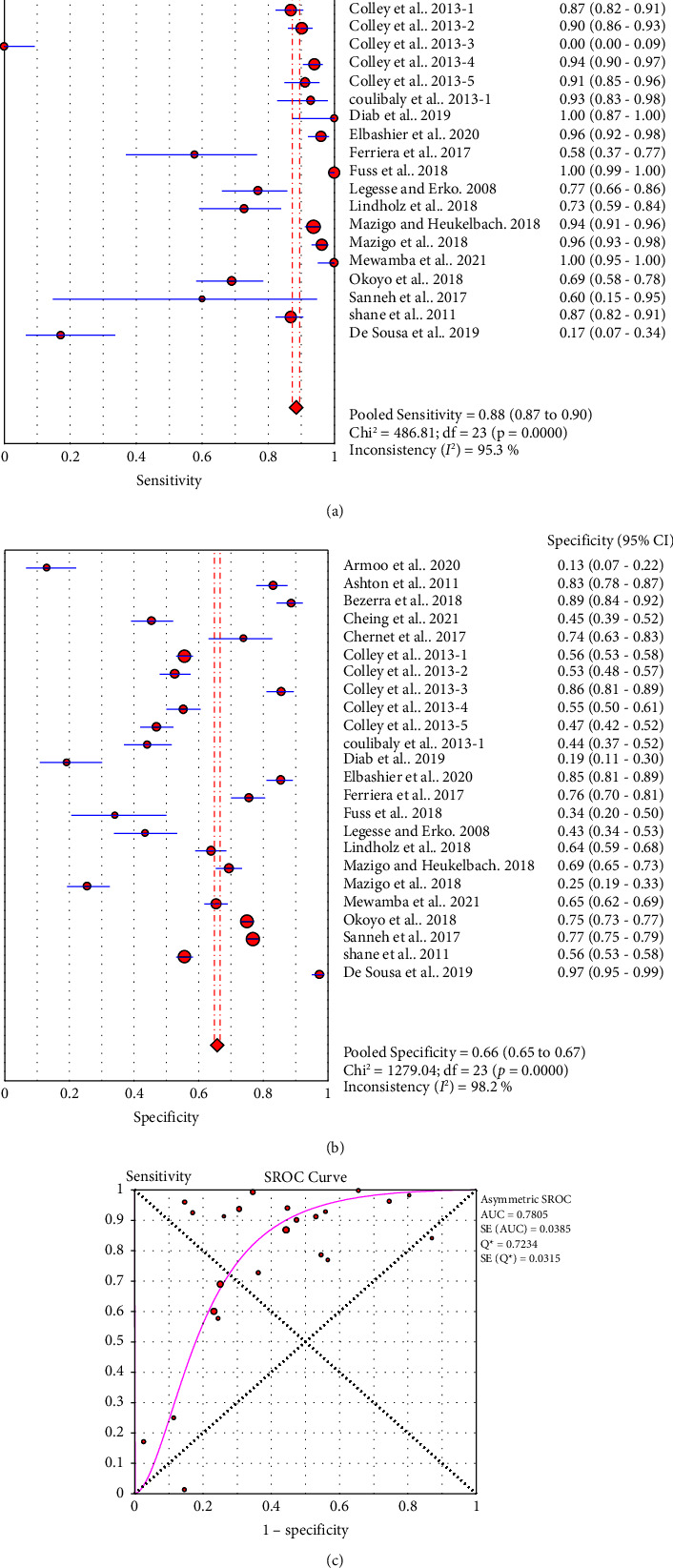
Sensitivity (a), specificity (b), and SROC curve (c) of single POC-CCA compared to KK reference standard (*n* = 24).

Twenty-four studies assessed the accuracy of a single POC-CCA test as compared to single KK reference test (duplicate 41.7 mg stool smear). The pooled sensitivity was high (0.88, 95% CI: 0.87–0.90), but the specificity was low (0.66, 95% CI: 0.65–0.67). The SROC curve showed an AUC of 0.7805 with standard error of 0.0385. This reveals that single POC-CCA test fairly discriminates infected participants from those without *Schistosoma* infection (Figure 4).

#### 3.3.2. POC-CCA versus KK with Different Number of Samples Examined

Simultaneous increase in the number of urine and stool samples collected at different days results in a slight increase in the sensitivity (0.88 versus 0.90) but a significant decrease in the specificity (0.66 versus 0.53) of POC-CCA test as compared to examination of single urine and stool samples. The pooled DOR is 11.44 (95% CI: 6.00–21.84) and the AUC was 0.8525 (data not shown). A few other studies collected different number of urine and stool samples for evaluation of POC-CCA. Pooled performances of each combination are presented in Table 2.

In studies comparing single POC-CCA and KK, the pooled LR+ was 2.10 (95% CI: 1.79–2.46). This means that the probability of POC-CCA test being positive among *S. mansoni* patients is 2.1 times higher than the probability of getting positive POC-CCA result among noninfected participants. The LR− in the same pair of tests was 0.22 (95% CI: 0.10–0.50), i.e., in participants with negative POC-CCA result, the probability of being infected decreases by 22% as compared to those tested positive by POC-CCA. The odds of getting a POC-CCA positive result among *S. mansoni* infected participants were 9.46 (95% CI: 6.03–14.85) (Table 3).

#### 3.3.3. Accuracy of POC-CCA versus PCR

**Figure 5:**
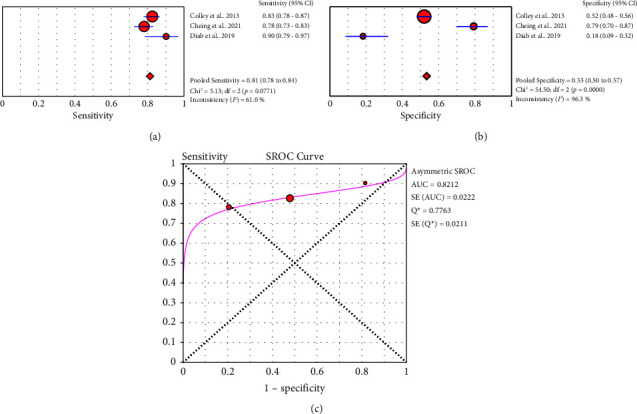
Sensitivity (a), specificity (b), and SROC curve (c) of single POC-CCA compared to PCR reference standard (*n* = 3).

Three studies reported the accuracy of POC-CCA against PCR (Table 4). The sensitivity of POC-CCA is low (0.81, 95% CI: 0.78–0.84), but the test agreement was good (AUC = 0.8212) (Figure 5).

## 4. Discussion

This systematic review assessed the accuracy of POC-CCA test for the diagnosis of *S. mansoni* infection using stool KK and PCR as reference standards. A similar review was published previously [47]; however, the authors did not consider the more sensitive PCR as a reference test. Furthermore, the quality of included studies was not assessed in the previous review, making the strength of evidence uncertain. Moreover, more studies are published since the previous review was completed and we were interested to produce a comprehensive review by including recently published studies.

All the included studies were cross-sectional where urine and stool samples were simultaneously collected and processed by POC-CCA (urine), KK (stool), and PCR (urine or stool). This might minimize the strength of evidence because participants were screened without controlling factors responsible to affect test performances of both the index and reference tests. All the included studies have compared accuracy of POC-CCA with KK while only 3 studies compared the index test with PCR. As can be observed from the risk of bias and applicability concern graph (Figure 2), majority of the included studies are with good quality. Therefore, the evidence generated in this review is strong enough to infer.

Comparison of POC-CCA with KK regardless of the number of urine and stool samples examined revealed good accuracy (sensitivity of 0.86 and specificity of 0.66). However, CCA excretion in urine shows day to day fluctuation [22] that pooled accuracy from studies examining different number of urine does not provide strong evidence. Similarly, the number of stool samples collected and the number of smears examined from each sample determine the performance of the reference KK test [11, 12, 22, 25, 31]. Therefore, we have done a subgroup analysis based on the frequency of urine and stool samples collected and examined. *Q*^2^ and *pvalues* presented in Figures 3, 4, and 5 imply that there is significant heterogeneity among studies. As a result, we used the random effect model during estimation of summary measures.

Subgroup analysis results show that single urine POC-CCA test has a better performance than single stool KK (Figure 4). This is in line with results of previous review where the pooled sensitivity and specificity were 0.90 (95% CI: 0.84–0.94) and 0.56 (95% CI: 0.39–0.71), respectively [47]. A good test should have high sensitivity as well as specificity. But in reality, as the sensitivity increases, specificity decreases for many tests. In most cases, sensitivity and specificity values of 0.90 or above are considered as high. However, what qualifies for “high” sensitivity or specificity varies by test. A high sensitivity is clearly important where the test is used to identify a serious but treatable disease or for screening of large population [48]. Hence, for *Schistosoma* infection, sensitivity will be the primary concern.

Accuracy of a test is estimated by simultaneous consideration of both sensitivity and specificity. For this purpose, we plotted SROC graph using values of sensitivity (true positive rate) on the *y*-axis against false positive rate (1−specificity) on the *x*-axis at different threshold points. The AUC represents the overall accuracy of the test. Accordingly, single urine POC-CCA has a test accuracy of 0.7805 as evaluated by single stool KK, interpreted as fair accuracy [18]. The reference test (KK) has its own limitation as it is poorly sensitive that the accuracy would be higher if a more sensitive reference test would have been used. The POC-CCA accuracy was higher when more than one urine sample was examined (AUC > 0.8) as shown in Table 2. POC-CCA shows low sensitivity (0.81, 95% CI: 0.78–0.84) as evaluated against PCR reference test, but the overall accuracy was good (AUC = 0.8212). However, this should be interpreted with caution because the evidence was generated only from three studies.

Performance of the POC-CCA test was also evaluated in terms of LR+, LR−, and DOR as shown in Table 3. For all combinations of samples where KK was the reference test, the LR + falls within 2.07–3.85, implying use of POC-CCA has a small increase in detecting infected participants than KK. This might be due to the low sensitivity of the reference KK which contributes for high FP rate by the index POC-CCA test. Index tests with LR+ of 5–10 and >10 are said to bring a moderate and large increase, respectively, in detecting patients [49]. Likewise, LR− is the probability that patients with the disease are likely to have a negative test result (FN) compared to participants without the disease. In the present review, the LR−was 0.22 (95% CI: 0.10–0.50) in the subgroup of studies targeting single POC-CCA and single KK. This is interpreted as use of POC-CCA decreases FN results by 22%. Due to its low detection threshold (20–50 eggs per gram of stool), KK is expected to miss significant number of patients who might be tested positive by CCA. Index tests with LR− > 0.1 are considered to be significant in decreasing the FN rate [49]. Diagnostic odds ratio is the ratio of LR+ and LR− and indicates the impact of an index test in decreasing both the FP and FN rates. The POC-CCA was found good in this respect as compared to KK (DOR ≥ 10) [49].

## 5. Limitations

We have excluded studies which assessed the accuracy of POC-CCA but reported sensitivity and specificity without providing data on TP, TN, FP, and FN. We did not conduct any modeling to create a good reference method. The other important limitation in the present review is that we did not stratify studies based on the endemicity level or intensity of infection, as we were unable to get complete data in most studies.

## 6. Conclusion

The commercially available POC-CCA test has higher sensitivity than KK and may serve as routine diagnostic alternative for disease diagnosis, mapping, and monitoring of interventions. However, the results should be interpreted with caution as we did not consider variations in disease endemicity and intensity of infection. The KK test itself is with poor sensitivity that evaluation of POC-CCA using more sensitive molecular reference standard tests is recommended. Therefore, we recommend well-designed multicenter accuracy studies involving diverse endemicity settings and geographic locations.

## Figures and Tables

**Table 1:** Characteristics of included studies for analysis of POC-CCA accuracy versus KK (*n* = 37).

Reference	Country	Sample size	Age group	CCA positive	KK positive	TP	FP	FN	TN	Samples tested	
										CCA	KK
[19]	Sudan	500	SAC	151	111	110	41	1	348	2	2
[20]	Ghana	148	PSAC	127	63	53	74	10	11	1	1
[21]	Sudan	373	SAC	154	111	111	43	9	210	1	1
[40]	Brazil	297	>2 yrs	30	4	1	29	3	225	1	1
[41]	Brazil	127	All	84	62	53	31	9	34	3	3
[22]	Tanzania	404	SAC	303	172	155	148	17	84	3	3
[23]	Kenya	357	All	223	117	92	131	25	109	1	1
[46]	Eritrea	107	Adults	43	23	21	22	2	62	1	1
[24]	Kenya	1845	SAC	921	279	231	664	35	833	1	1
[24]	Cameroon	733	SAC	456	281	247	208	27	231	1	1
[24]	Côte d'Ivoire	607	SAC	276	291	0	42	38	249	1	1
[24]	Ethiopia	620	SAC	409	267	251	158	16	195	1	1
[24]	Uganda	500	SAC	313	125	114	199	11	176	1	1
[25]	Côte d'Ivoire	323	SAC	106	271	93	13	58	159	1	3
[25]	Côte d'Ivoire	332	SAC	150	165	121	29	44	138	3	3
[25]	Côte d'Ivoire	444	SAC	144	171	142	2	129	171	1	3
[26]	Côte d'Ivoire	242	SAC	156	56	52	104	4	82	1	1
[26]	Côte d'Ivoire	242	PSAC	185	56	53	132	3	54	2	1
[27]	Uganda	82	PSAC	51	37	37	14	7	22	1	2
[28]	Egypt	100	All	86	27	27	59	0	14	1	1
[29]	Sudan	489	SAC	214	175	168	46	7	268	1	1
[31]	Ethiopia	620	SAC	439	329	313	126	16	175	3	3
[42]	Brazil	300	All	82	26	15	67	11	207	1	1
[30]	Tanzania	297	SAC	282	253	253	29	0	15	1	1
[32]	Uganda	446	All	346	395	337	9	58	39	1	3
[16]	Ethiopia	184	SAC	120	67	60	60	18	46	1	1
[43]	Brazil	580	1–17 yrs	187	55	40	147	15	259	1	1
[33]	Tanzania	979	All	592	463	434	158	29	358	1	1
[34]	Tanzania	419	All	365	242	233	132	9	45	1	1
[35]	Cameroon	759	SAC	309	71	71	238	0	450	1	1
[36]	Kenya	3560	SAC	479	87	60	419	27	1255	1	1
[37]	Gambia	1954	SAC	456	5	3	453	2	1496	1	1
[38]	Kenya	426	SAC	264	187	231	664	35	833	1	1
[44]	Brazil	141	>1 yr	32	15	13	19	2	107	2	1
[45]	Brazil	372	SAC	15	35	6	9	29	328	1	1
[39]	Cameroon	625	SAC	316	381	322	94	59	150	1	3
[39]	Cameroon	625	SAC	510	381	355	145	26	99	3	3

**Table 2:** Subgroup analysis of different combinations of urine and stool samples.

Samples	Reference	Sen	(95% CI)	Spe	(95% CI)	AUC
3 urine versus 3 stool	[41]	0.90	0.85–0.94	0.36	0.30–0.43	
	[25]	0.73	0.66–0.80	0.83	0.76–0.88	
	[32]	0.95	0.92–0.97	0.58	0.52–0.64	
	[39]	0.93	0.90–0.96	0.41	0.34–0.47	
	Pooled	0.90	0.88–0.92	0.53	0.49–0.56	0.8524

1 urine versus 3 stool						
	[22]	0.86	0.74–0.93	0.52	0.40–0.65	
	[25]	0.52	0.46–0.59	0.99	0.96−0.10	
	[31]	0.55	0.81–0.88	0.81	0.67–0.91	
	[39]	0.85	0.81–0.88	0.62	0.55–0.68	
	Pooled	0.77	0.74–0.80	0.74	0.70–0.78	0.8550

2 urine versus 1 stool	[26]	0.95	0.85–0.99	0.29	0.23–0.36	
	[43]	0.87	0.60–0.98	0.85	0.78–0.91	
	Pooled	0.93	0.84–0.98	0.52	0.46–0.57	

**Table 3:** Likelihood and diagnostic odds ratio for accuracy of POC-CCA.

Tests compared	LR+ (95% CI)	LR− (95% CI)	DOR (95% CI)
1 CCA versus 1 KK	2.10 (1.79–2.46)	0.22 (0.10–0.50)	9.46 (6.03–14.85)
3 CCA versus 3 KK	2.07 (1.48–2.89)	0.19 (0.10–0.35)	11.44 (6.00–21.84)
2 CCA versus 1 KK	2.72 (0.61–12.23)	0.17 (0.07–0.40)	15.05 (3.01–75.38)
1 CCA versus 3 KK	3.85 (1.88–7.88)	0.28 (0.16–0.49)	16.55 (6.47–42.33)
CCA versus PCR	1.87 (1.10–3.19)	0.31 (0.25–0.38)	5.89 (2.47–14.00)

**Table 4:** Characteristics of included studies for analysis of POC-CCA accuracy versus PCR (*n* = 3).

Reference	Country	Sample size	Age group	CCA positive	PCR positive	TP	FP	FN	TN
[23]	Kenya	357	All	223	260	203	20	57	77
[24]	5 African countries	1845	SAC	539	318	251	288	53	313
[28]	Egypt	100	All	86	51	46	40	5	9

## Data Availability

Template data collection forms; data extracted from included studies; data used for all analyses; and the review protocol are available from the corresponding author.

## Abbreviations

AUC: Area under the curve
DOR: Diagnostic odds ratio
FN: False negative
FP: False positive
KK: Kato–Katz
LR: Likelihood ratio
MDA: Mass drug administration
PCR: Polymerase chain reaction
POC-CCA: Point‐of‐care circulating cathodic antigen
PSAC: Preschool-aged children
QUADAS-2: Quality Assessment of Diagnostic Accuracy Studies 2
SAC: School-aged children
SROC: Summary receiver operating characteristic
TN: True negative
TP: True positive
WHO: World Health Organization.
